# Diagnostic yield of cerebrospinal fluid analysis in status epilepticus: an 8-year cohort study

**DOI:** 10.1007/s00415-021-10447-3

**Published:** 2021-03-05

**Authors:** Tolga D. Dittrich, Sira M. Baumann, Saskia Semmlack, Gian Marco De Marchis, Sabina Hunziker, Stephan Rüegg, Stephan Marsch, Sarah Tschudin-Sutter, Raoul Sutter

**Affiliations:** 1 Department of Intensive Care Medicine, University Hospital Basel, Basel, Switzerland; 2 Department of Neurology, University Hospital Basel, Basel, Switzerland; 3 Medical Faculty of the University of Basel, Basel, Switzerland; 4 Department of Clinical Research, University of Basel, Basel, Switzerland; 5 Department of Medical Communication and Psychosomatic Medicine, University Hospital Basel, Basel, Switzerland; 6 Division of Infection Diseases and Hospital Epidemiology, University Hospital Basel, Basel, Switzerland

**Keywords:** Status epilepticus, Intensive care, Neurocritical care, Lumbar puncture, Cerebrospinal fluid

## Abstract

**Background:**

We investigate the frequency and diagnostic yield of cerebrospinal fluid (CSF) analysis in adult patients with status epilepticus (SE) and its impact on the outcome.

**Methods:**

From 2011 to 2018, adult patients treated at the University Hospital Basel were included. Primary outcomes were defined as the frequency of lumbar puncture and results from chemical, cellular, and microbiologic CSF analyses. Secondary outcomes were differences between patients receiving and not receiving lumbar puncture in the context of SE.

**Results:**

In 18% of 408 patients, a lumbar puncture was performed. Of those, infectious pathogens were identified in 21% with 15% detected ± 24 h around SE diagnosis. 74% of CSF analyses revealed abnormal chemical or cellular components without infectious pathogens. Screening for autoimmune diseases was only performed in 22%. In 8%, no or late (i.e., > 24 after SE diagnosis) lumbar puncture was performed despite persistent unknown SE etiology in all, transformation into refractory SE in 78%, and no recovery to premorbid neurologic function in 66%. Withholding lumbar puncture was associated with no return to premorbid neurologic function during hospital stay independent of potential confounders. Not receiving a lumbar puncture was associated with presumed known etiology and signs of systemic infectious complications.

**Conclusions:**

Withholding lumbar puncture in SE patients is associated with increased odds for no return to premorbid neurologic function, and CSF analyses in SE detect infectious pathogens frequently. These results and pathologic chemical and cellular CSF findings in the absence of infections call for rigorous screening to confirm or exclude infectious or autoimmune encephalitis in this context which should not be withheld.

**Supplementary Information:**

The online version contains supplementary material available at 10.1007/s00415-021-10447-3.

## Background

Status epilepticus (SE) is a neurologic emergency with high morbidity and mortality [23, 25, 27]. Since the etiology has a significant if not the most important effect on survival [23, 26] and identification of the underlying cause guides treatment, rapid and reliable diagnostic measures and workup are essential. While important investigations by means of anamnesis, physical and laboratory examination, as well as neuroimaging may uncover underlying SE etiologies, patients with unremarkable exams represent great challenges for the treating team. In these situations, every effort must be made to identify the cause of SE.

Encephalitis represents an important SE etiology that may come with normal or discrete and unremarkable neuroimaging and otherwise rather non-specific anamnesis, physical status, and laboratory findings. Although the detection of infectious pathogens in the cerebrospinal fluid (CSF) is the “gold standard” for the diagnosis of infectious (meningo-)encephalitis [6], CSF analyses are not consistently recommended in international guidelines regarding SE. While the Neurocritical Care Society Status Epilepticus Committee suggests considering the performance of a lumbar puncture in such clinical scenarios [4], CSF analyses are neglected by the guidelines of the European Federation of Neurological Societies [15] and the most recent evidence-based guideline on the treatment of convulsive SE in children and adults of the American Epilepsy Society [11]. As infectious (meningo-)encephalitis encompasses many different viral and bacterial infections of the central nervous system (CNS) [6] and accounts for up to 10% of SE etiologies (not accounting for missed or underdiagnosed cases) [28, 35], the question arises whether or not CSF analyses should be labeled as mandatory with persistent unknown etiology, as already recommended for the diagnosis of new-onset refractory SE (NORSE) or cryptogenic NORSE [13, 34]. This comes with uncertainty once CSF is analysed regarding the extent to which chemical and cellular changes may be caused by SE per se and not by underlying infectious or autoimmune diseases. Despite these worrisome facts and uncertainties, studies in this context are lacking.

We aimed to investigate the frequency and diagnostic yield of cerebrospinal fluid analysis in adult patients with SE.

## Materials and methods

This observational study was performed at the University Hospital of Basel, a Swiss tertiary academic medical care center. The STROBE-guidelines were followed to enhance the quality and standardization for the reporting of observational studies [32]. The Swiss ethics committee (Ethikkommission Norwest- und Zentralschweiz) approved the study in compliance with the ethical standards laid down in the 1964 Declaration of Helsinki and its later amendments and waived patients’ consent.

### Data collection

Data analysed in this study are part of the ongoing **ST**atus **EP**ilepticus **U**nicenter **P**opulation (**STEP UP**) study (Clinicaltrials.gov No. NCT04348318). From January 1st, 2011 to December 31st, 2018, clinical, laboratory, and epileptologic data of all consecutive adult SE patients (≥ 18 years of age) were assessed with the digital institutional electroencephalographic (EEG) and medical databases. Patients with SE in the context of acute hypoxic-ischemic encephalopathy were excluded.

The following clinical data were collected: age, sex, etiology (categorized as potential non-fatal and fatal as defined elsewhere [17]), quantified comorbidities by the Charlson Comorbidity Index [5], intubation and duration of mechanical ventilation, the use of antiseizure drugs and continuously administered anesthetics, and complications emerging during SE were noted. In addition, the time of performance of a lumbar puncture and results from chemical and microbiologic analyses of the cerebrospinal fluid were assessed. Severity of SE was quantified by the most frequently validated Status Epilepticus Severity Score (STESS) as previously described [18, 19, 22, 24]. Performance and types of neuroimaging [i.e., cerebral magnetic resonance imaging (MRI) and/or computed tomography (CT)] were assessed. Pathologic findings, as described after the images have been examined by two radiologists were noted. Finally, the length of the intensive care unit (ICU) and hospital stay were recorded.

### Classification of SE and SE duration

Classification of SE followed the guidelines of the task force on the classification of SE of the International League Against Epilepsy [29]. SE types were categorized as focal nonconvulsive SE without coma with or without altered consciousness and absence SE, SE with motor symptoms (including myoclonic and convulsive forms), and nonconvulsive SE with coma.

SE duration was defined as the time span between the clinical and/or EEG evidence of seizure onset and the time-point at which seizure termination was proven by EEG*.* SE duration is expressed as a 12 h approximation, as two different EEG recording strategies were used, such as continuous EEG or spot EEGs of ≥ 30 min every 12 h. Continuous EEG was consistently used for refractory SE.

### Detection of infectious pathogens, chemical and cellular CSF analyses

The microbiologic workup and the diagnoses of (meningo-)encephalitis were systematically established according to the pre-established frameworks [6, 7]. The diagnostic algorithms for infectious (meningo-)encephalitis of the consensus statement of the international encephalitis consortium were followed [30] and based on the detection of viral ribonucleic acid (RNA) or deoxyribonucleic acid (DNA) of infectious pathogens with PCR in the CSF. The diagnosis of bacterial (meningo-)encephalitis was established with the microscopic detection of Gram-stained infectious pathogens in the CSF, the detection of aerobic and anaerobic bacterial cultures for 6 days, and/or PCR. Due to local conditions, two additional diagnostic procedures were applied. First, the diagnosis of *Borrelia burgdorferi* infection was established according to the national guidelines by the detection of intrathecal antibodies [8]; second, the diagnosis of tick-borne encephalitis (“Frühsommer” Meningoencephalitis, FSME) was diagnosed with positive serology [31].

Chemical analyses, such as the CSF concentrations of proteins, glucose (including the glucose ratio CSF/serum), lactate, IgG, IgA, and IgM Indexes and quotients and Reiber diagrams, oligoclonal bands, and screening for autoantibodies (including antibodies against *N*-methyl-d-aspartate [NMDA]-receptor, voltage-gated-kalium-channels [VGKC], and glutamic acid decarboxylase (GAD)) were assessed. In addition, information from screening for paraneoplastic antibodies including anti-Hu (ANNA-1)-, anti-Yo (PCA-1)-, anti-Ri (ANNA-2)-, anti-CV2 (CRMP5)-, anti-Ma1 (PNMA1)-, anti-Ma2/Ta (PNMA2)-, and anti-amphiphysin-antibodies were collected. Screening for autoantibodies and paraneoplastic antibodies was not restricted to the CSF and was also performed serum samples.

During the study period, all CSF were analyzed regarding cellular and chemical characteristics including levels of lactate, glucose, proteins, Gram staining, and bacterial cultures. Further analyses regarding immunoglobulin synthesis, PCR for detection of infectious pathogens, screening for autoantibodies were not standardized, as during the study period, no institutional algorithms were in place. Hence, further analyses, including the performance of IgG, IgA or IgM quotient diagrams and Reiber diagrams, PCRs, and screening for autoantibodies were performed at the discretion of the treating and/or consulting physicians.

### Outcomes

Primary outcomes were defined as the frequency of lumbar puncture and results from chemical and microbiologic cerebrospinal fluid analyses.

Differences between patients receiving and not receiving lumbar punctures and associations between lumbar puncture and return to premorbid neurologic function during hospital stay were considered secondary outcomes.

### Statistics

Patients were categorized into patients with and without lumbar puncture during their hospital stay. Univariable comparison of patients with and without lumbar puncture was performed by the Chi-square test or the Fisher’s exact test. For continuous variables, the Shapiro–Wilk test was used to distinguish between normally and not normally distributed variables. Normally distributed variables were analyzed with the Student’s *t* test, whereas variables violating the normal distribution were analyzed with the Mann–Whitney *U* test.

Univariable logistic regression models were applied to calculate odds ratios for the associations of not having received a lumbar puncture with no return to premorbid neurologic function. Multivariable logistic regression model was subsequently performed to adjust the association between no performance of lumbar puncture and no return to premorbid neurologic function for potential confounders including potential fatal etiology, STESS, duration of SE, the use of continuous anesthetics, and systemic infectious complications (as detected in the univariable comparison between patients receiving and not receiving lumbar punctures).

Two-sided *p*-values ≤ 0.05 were considered significant. Statistical analysis was performed with STATA^®^16.1 (Stata Corp., College Station, TX, USA).

### Data availability

Anonymized data are available from the corresponding author and will be shared on reasonable request from any qualified investigator.

## Results

**Fig. 1:**
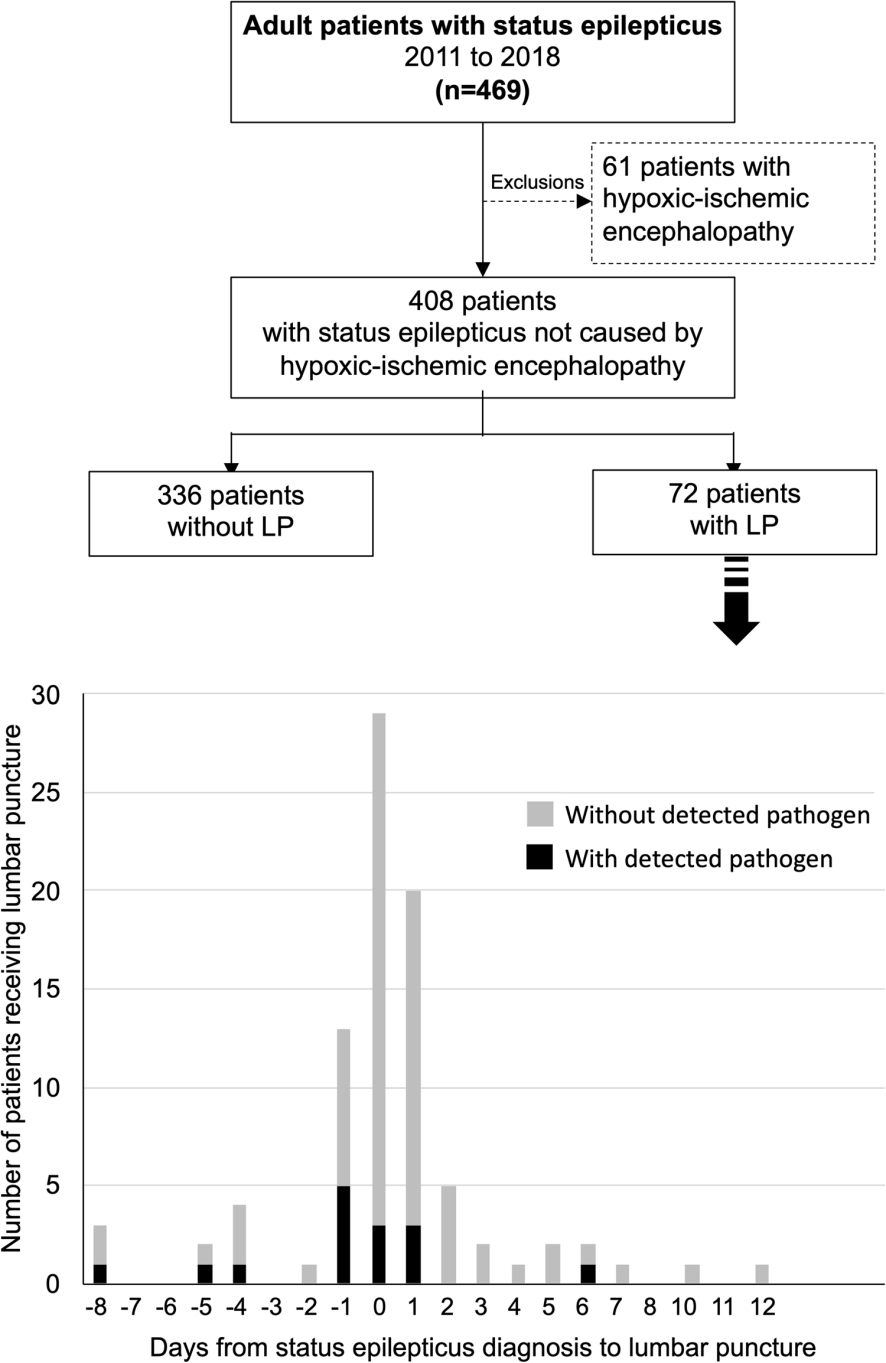
Flow chart and performance of lumbar puncture in patients with status epilepticus. *LP* lumbar puncture

From January 2011 to December 2018, 469 adult patients were treated for SE in the University Hospital Basel. Of those, 61 (13%) developed SE from hypoxic-ischemic brain injury following cardiorespiratory arrest and were excluded from the study (Fig. 1).

### Primary outcomes

**Fig. 2:**
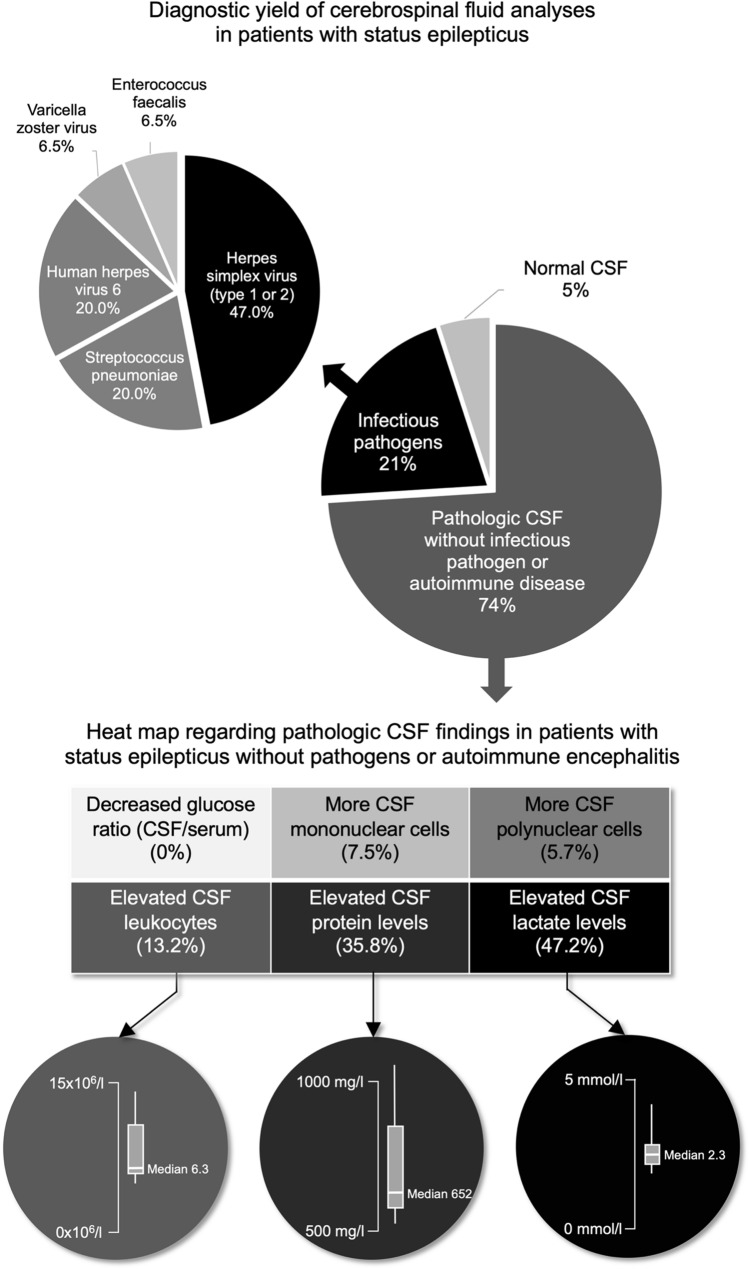
Diagnostic yield of cerebrospinal fluid analyses in patients without infectious pathogens or autoimmune encephalitis. *CSF* cerebrospinal fluid

Of the remaining 408 adult patients with SE, lumbar puncture was performed during their hospital stay in 72 patients (17.6%) within a median of 0 days [interquartile range (IQR) 0–1] from SE diagnosis. Of those, infectious pathogens were identified in 15 patients (20.9%), with 11 being detected ± 24 h around SE diagnosis (73.3% of detected infections). Detailed information regarding the detected infectious pathogens and pathologic chemical and cellular components of the CSF in patients without infectious pathogens or identified autoimmune disease are presented in Fig. 2. Of all 72 CSF analyzed, 53 (74%) showed abnormal increases of either chemical (i.e., protein and/or lactate concentrations) or cellular (i.e., mono- and/or polynuclear leukocytes) components but no infectious pathogens and no evidence of autoimmune disease involving the nervous system. In patients receiving lumbar puncture, neuroimaging was performed in 57 (79%), including 43 (60%) MRI and 57 (79%) CT. Of those, signs of possible cerebral inflammation were found in 3 (5%), signs of acute ischemic strokes in 2 (3%), and signs of brain tumors in 4 (7%) patients.

**Fig. 3:**
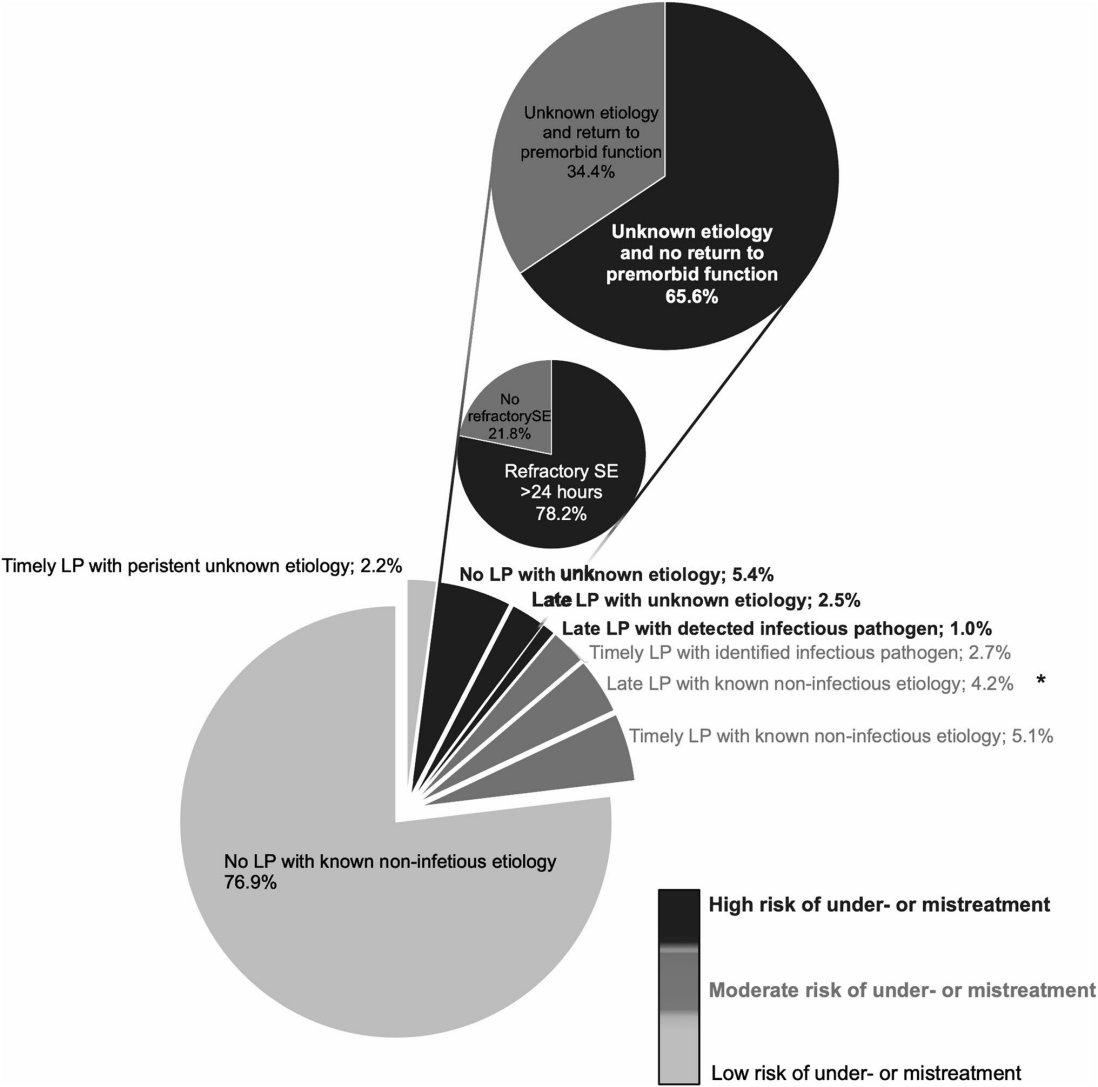
Performance of lumbar puncture in patients with status epilepticus. *LP* lumbar puncture, *late LP* lumbar puncture after 24 h following the diagnosis of SE. *Of patients with late LP with identified non-infectious etiology, one had NMDA encephalitides. In all others, SE etiology was known prior to LP

Figure 3 presents further details regarding the performance of timely (i.e., within 24 h after SE diagnosis) and late (i.e., after 24 h after SE diagnosis) lumbar puncture in patients with different constellations regarding known or unknown etiologies. Infectious pathogens were detected in 21% of patients with CSF analyses, with most pathogens being *herpes simplex virus type 1 or 2*, followed by *streptococcus pneumoniae.*

Remarkably, in 32 patients (7.9%) no or late lumbar puncture was performed despite having a persistent unknown etiology of SE in 100% and not having complete recovery to their premorbid neurologic function in 65.6% during their hospital stay. Of those 32 patients, 25 (78.2%) SE was refractory with a duration of more than 24 h, and nine patients developed cryptogenic NOSRE. Of those, lumbar puncture was performed in only three (30% of NOSRE) without detection of infectious pathogens, no oligoclonal bands or an increased IgG index, and only two (22% of NOSRE) patients were screened for anti-*N*-methyl-d-aspartate (NMDA)-receptor antibodies in the CSF and serum revealing negative results. In our total cohort, two patients were diagnosed with having developed SE from anti-NMDA-receptor antibody encephalitis in whom the antibodies were detected in their serum. Screening for oligoclonal bands and IgG index, IgG and Reiber diagrams were performed in 16 (22% of patients with lumbar puncture) patients with all having no elevated oligoclonal bands and none having elevated IgG. Antibody screening of the CSF and serum was also only performed in nine patients (13% of patients with lumbar puncture) regarding anti-NMDA-receptor antibodies, including additional screening for anti-voltage-gated-kalium-channels (VGKC)-antibodies in 8 and one for anti-glutamic acid decarboxylase (GAD) antibodies. Screening for paraneoplastic antineuronal antibodies in the serum was performed in 5 patients, including anti-Hu (ANNA-1)-, anti-Yo (PCA-1)-, anti-Ri (ANNA-2)-, anti-CV2 (CRMP5)-, anti-Ma1 (PNMA1)-, anti-Ma2/Ta (PNMA2)-, and anti-amphiphysin-antibodies. Detailed information regarding the diagnostic CSF-workup is presented in the online supplemental Table 1.

### Secondary outcomes

Comparison of clinical characteristics, treatment, course, and outcome of patients with and without lumbar puncture are presented in Table 1. In patients with lumbar puncture, the etiology of SE was unknown more frequently, SE was less frequently convulsive, duration of SE and ICU treatment was longer, and infections were detected more often. Uni- and multivariable analyses regarding the association between no performance of lumbar puncture and outcome are presented in Table 2 (upper part). Univariable analysis revealed no significant association between withholding lumbar puncture and no return to premorbid neurologic function. When adjusting for potential confounders as detected in the comparison between patients with and without lumbar puncture (as shown in Table 1), withholding lumbar puncture was found to be an independent association with no return to premorbid neurologic function.

**Table 1:** Characteristics of patients with status epilepticus with and without lumbar puncture (*n* = 408)

Characteristics	Total cohort (*n* = 408)		Patients without lumbar puncture (*n* = 336)		Patients with lumbar puncture (*n* = 72)		*p*-value
Demographics and admission characteristics							
Age (years; median, IQR)	67.2	54–78	68.2	54–78	65.6	54–74	0.325
Sex (female; *n*, %)	198	48.5	160	47.6	38	52.8	0.427
Clinical features							
SE etiology (*n*, %)							
Known etiology	367	89.9	314	93.5	53	73.6	**< 0.001**
Confirmed potential fatal etiology (not mutually exclusive)	134	32.8	109	32.4	25	34.7	0.708
Fast growing brain tumors	34	8.3	30	8.9	4	5.6	
Acute intracranial hemorrhage	41	10.0	39	11.6	2	2.8	
Infectious (meningo-)encephalitis	15	20.8	0	0.0	15	20.8	
Acute ischemic stroke	16	3.9	14	4.2	2	2.8	
Acute severe traumatic brain injury	12	2.9	12	3.6	0	0.0	
Acute autoimmune encephalitis	2	0.5	1	0.3	1	1.4	
Septic encephalitis	5	1.2	4	1.2	1	1.4	
Others	9	2.2	7	2.1	2	2.8	
Unknown etiology	41	10.0	22	6.5	19	26.4	**< 0.001**
SE type# (*n*, %)							
Focal NCSE without coma	167	40.9	140	41.7	27	37.5	0.514
With altered consciousness	145	35.5	123	36.6	22	30.6	
Without altered consciousness	22	5.4	17	5.1	5	6.9	
SE with motor symptoms (convulsive, myoclonic)	129	31.6	114	33.9	15	20.8	**0.034**
Convulsive SE	87	21.3	77	22.9	10	13.9	
Myoclonic SE	42	10.3	37	11.0	5	6.9	
NCSE with coma	112	27.5	82	24.4	30	41.7	**0.003**
NCSE with coma (non-subtle)	80	19.6	60	17.9	20	27.8	
Subtle SE	32	7.8	22	6.6	10	13.9	
Charlson Comorbidity Index* (median, IQR)	2	1–4	2	1–5	2	1–4	0.231
STESS (median, IQR)**	3	2–4	3	1–4	3	2–4	0.052
SE duration (days; median, IQR)	1	0.5–2	1	0.5–1	1.5	0.75–3	**< 0.001**
Treatment characteristics during SE							
In-hospital treatment (days; median, IQR)	13	6–22	12	5–21.5	14.5	8.5–24.5	0.051
ICU treatment (days; median, IQR)	2	1–6	2	0–5	4	2–10	**0.002**
Patients with benzodiazepines (*n*, %)	403	98.8	333	99.1	70	97.2	
Patients with second-line antiseizure drugs (*n*, %)	396	97.1	324	96.4	72	100.0	
Number of non-anesthetic antiseizure drugs (median, IQR)	3	2–4	3	2–4	3	2–4	0.319
Mechanical ventilation (*n*, %)	145	35.5	117	34.8	28	38.9	0.513
Duration of mechanical ventilation (days; median, IQR)	5	2–13	5	2–11	9	4–14	0.216
Complications during SE (*n*, %)							
Infections	75	18.4	54	16.1	21	29.2	**0.009**
Arterial hypotension requiring vasopressors	44	10.8	34	10.1	10	13.9	0.349
Cardiorespiratory resuscitation	4	1.0	3	0.9	1	1.4	
Multiorgan failure	3	0.7	3	0.9	0	0.0	
Outcomes (*n*, %)							
No return to premorbid neurologic function at discharge (incl. death)	237	58.1	198	58.9	39	54.2	0.457
Death at hospital discharge	30	7.4	25	7.4	5	6.9	1.000

*CNS* central nervous system, *SE* status epilepticus, *NCSE* nonconvulsive status epilepticus, *CSE* convulsive status epilepticus, *ICU* intensive care unit, *IQR* interquartile range

*Charlson Comorbidity Index [5]

**STESS = Status Epilepticus Severity Score (Range 0–6) [18, 19, 22]

^#^SE types according to the task force of the International League Against Epilepsy (ILAE) [29]

**Table 2:** Uni- and multivariable analyses regarding the associations with no return to premorbid neurologic function and no performance of lumbar puncture

	Univariable analyses			Multivariable analyses*		
	OR	95% CI	*p*-value	OR	95% CI	*p*-value
No return to premorbid neurologic function						
No performance of lumbar puncture	1.21	0.73–2.03	0.458	2.25	1.18–4.27	**0.013**
Potential fatal etiology	2.51	1.61–3.92	**< 0.001**	1.83	1.09–3.07	**0.023**
Unknown etiology	1.14	0.59–2.21	0.693	1.16	0.51–2.66	0.725
STESS	1.76	1.52–2.04	**< 0.001**	1.79	1.52–2.11	**< 0.001**
Use of continuous anesthetics	2.77	1.69–4.52	**< 0.001**	2.36	1.33–4.19	**0.003**
Duration of SE (per day)	1.25	1.10–1.43	**0.002**	1.22	1.08–1.39	**0.002**
Systemic infectious complications	1.95	1.13–3.35	**0.016**	0.97	0.51–1.86	0.928
No performance of lumbar puncture						
Known etiology#	5.12	2.59–10.09	**< 0.001**	5.79	2.79–12.01	**< 0.001**
SE with motor symptoms#	1.95	1.06–3.60	**0.032**	1.69	0.87–3.26	0.120
STESS#	0.85	0.73–0.99	**0.042**	0.92	0.78–1.08	0.302
Use of continuous anesthetics#	0.49	0.29–0.84	**0.009**	0.60	0.33–1.10	0.101
Duration of SE (per day)#	0.98	0.93–1.02	0.325	1.03	0.97–1.09	0.363
Systemic infectious complications#	0.46	0.26–0.84	**0.010**	0.47	0.24–0.91	**0.026**

*SE* status epilepticus, *STESS* Status Epilepticus Severity Score (Range 0–6) [18, 19, 22], *OR* odds ratio, *CI* confidence interval

^#^All variables that significantly differed in the comparison of Table 1

*Hosmer–Lemeshow goodness of fit test Chi^2^ 13.43; *p* = 0.100 indicating adequate model fit

**Hosmer–Lemeshow goodness of fit test Chi^2^ 6.33; *p* = 0.611 indicating adequate model fit

Further uni- and multivariable analyses revealed that known etiology and absence of infectious complications were independent associations with patients not receiving a lumbar puncture (Table 2, lower part).

## Discussion

Despite current recommendations regarding the management of status epilepticus [4], lumbar puncture was inconsistently performed in our patients in whom the other diagnostic steps have not clarified the etiology of SE and whose neurological condition has not fully recovered to premorbid status. Our study further revealed that infectious pathogens were identified frequently once CSF analysis was performed. The most commonly detected pathogens were *herpes simplex virus type 1 or 2*, *streptococcus pneumoniae,* and *human herpes virus 6*. While the latter seems surprising in adult patients, the fact that our institution treats a large number of immunosuppressed and hematologic patients is a possible explanation, as they are susceptible to infections with or reactivation of less common pathogens such as *human herpes virus 6* [33]. As most detected infectious encephalitides are potentially treatable, the underperformance of CSF analyses seems critical and may also be at least a partial explanation of why withholding a lumbar puncture was independently associated with increased odds for no return to premorbid neurologic function. Our results further indicate that presumed known etiology and the absence of infectious complications were independently associated with increased odds of not receiving a lumbar puncture. This indicates that clinicians are more likely to withhold lumbar puncture with the absence of clinical signs of infections, a critical decision especially in the context of viral or autoimmune encephalitis, which often emerges without clinical signs of inflammation. Although the association between not performing a lumbar puncture and outcome seems plausible at first glance, our results have to be interpreted with caution, as our analyses cannot exclude clinical scenarios in which clinicians had a supportive rationale for withholding diagnostic procedures, such as when the likelihood of an expected poor outcome is presumed to be high. Another scenario in which lumbar puncture is not performed with good reason is the presence of a cerebral mass with signs of increased intracranial pressure or brainstem compression on neuroimaging—a situation not described in the neuroimaging in any of our patients, not even in the patients with brain tumors and neuroimaging.

The underuse of CSF analyses may also be explained by the fact that during the study period no institutional guidelines regarding the performance of lumbar punctures were in place and that the guidelines of the Neurocritical Care Society only provide vague recommendations that lumbar puncture should be considered in specific cases, and most other SE guidelines do not even mention the lumbar puncture and CSF analyses as an important diagnostic step [11, 15]. Our finding that infectious pathogens were identified in every fifth patient in whom CSF was analyzed indicates that in SE patients without known etiology, infections of the CNS are frequent and may be underestimated due to the underuse of CSF analyses. This comes along with a high probability of underdiagnosed autoimmune diseases as screening for autoimmune diseases was not performed systematically in our patients with persistent unknown etiology of SE. Although it is unclear why current guidelines “toned down” or neglected the importance of CSF analyses, our results suggest that CSF analyses should be performed more frequently, since many patients have a persistent undetermined etiology of SE and lumbar puncture is safe if neuroimaging reveals no signs of increased intracranial pressure.

The fact that lumbar puncture was less frequently performed, especially in patients with convulsive SE, may be explained by the fact that during convulsions invasive diagnostic procedures put the patients at additional risks of injury in conjunction with unpredictable motor activity of the patient. However, as most patients were in refractory SE for more than 24 h and treated with the induction of deep coma, this assumption may only be a partial explanation.

Another important finding is the large proportion of abnormal chemical and/or cellular CSF components in patients in whom microbiologic workup did not detect infectious pathogens. To what degree these CSF findings were explained by the inflammation as a result of an excessive intracerebral increase of neurotransmitters to neurotoxic concentrations due to SE per se or by undetected or unsuspected autoimmune CNS disease remains unclear, as screening for autoantibodies was left to the discretion of the clinician and was not performed systematically. Although glucose CSF/serum ratios and lactate concentrations were mostly unremarkable, CSF protein levels and leukocyte counts were frequently and noticeably increased even without detectable infectious pathogens. In this context, it is important to mention that according to a recent systematic review there is concordance in the available literature to recommend increasing CSF total protein upper reference limits and to consider implementing age-adjusted values above 600 mg/L starting at age 50 [3]. However, when talking this into account, only 6 patients with a protein level above 500 mg/L without proof of infectious or autoimmune encephalitis were then still considered within the normal range.

During the last decade, CSF examination for non-infectious etiologies of SE has expanded, uncovering a number of autoimmune diseases that can be linked to the emergence of autoimmune encephalitis driven SE, and therapeutics in instances have been tailored for specific findings. Remarkably, only a very small number of patients in our cohort were screened for autoantibodies in the CSF, and diagnosis of anti-NMDA-receptor encephalitis was diagnosed in only two of our patients. Although information regarding the screening of antibodies in the patients' blood samples could not be assessed, as these analyses were performed in out-of-hospital test centers during the study period, underestimation and missed diagnosis is obvious, especially for patients with severe conditions including NOSRE in whom investigations regarding non-infectious CNS disorders are crucial, lumbar puncture was only performed in every third patient. This is worrisome, as brain MRI studies can be unremarkable in more than half of patients with autoimmune encephalitides [12, 20] and autoimmune encephalitis is be detected in the context of NOSRE in up to 20% in prior studies [10]. To reduce the risk of missing or underdiagnosing infectious or autoimmune encephalitis in patients with SE, the authors propose an algorithm for the decision of whether to perform a lumbar puncture or not in patients with SE (Online supplemental Fig. 1). If the etiology of SE remains unclear after routine chemical, cellular and microbiologic analyses of the CSF, antibody screening should be performed to uncover autoimmune encephalitis as recently outlined [12].

### Limitations

The single-center observational design limits the generalizability of this study. The fact, however, that the clinical characteristics in our population are similar to those in other SE studies, including age [1, 2, 9, 16, 21], outcome [9, 14], etiologies [2, 9, 14, 21], SE severity [2, 9], and types of SE [1, 14, 16] indicate that our cohort has several characteristics typical of other large SE cohorts.

Autoimmune diseases were not routinely screened in our institution, and most investigations are initiated upon suspicious patient's history and/or neuroimaging. Therefore, it remains unclear to what extent autoimmune diseases may have been missed and have at least partially explained the pathologic CSF findings. As our understanding regarding autoimmune encephalitis as a cause of SE has expanded in recent years, underestimation and missed diagnosis in this context is very likely, especially in the first years of our study. In addition, signs of activation of the immune system, such as the presence of plasmacells, large activated lymphocytes, or macrophages were not systematically documented in our institution. Another limitation comes from the fact that our study design does not allow any analyses regarding the number of patients receiving antimicrobials for suspected or proven CNS infection, as several patients were also treated for infectious complications outside the CNS and the differentiation in retrospect between antimicrobials only administered for one or the other is error prone and not possible in every patient. For the same reason, unfortunately, analyses regarding the effects of antimicrobial treatment on course and outcome would be subject to substantial bias.

Finally, the approximation of SE duration represents another limitation regarding potential underestimation of SE duration, especially with unwitnessed onset, mainly the case with nonconvulsive SE [27].

## Conclusions

Withholding lumbar puncture in SE patients is associated with increased odds for no return to premorbid neurologic function, and CSF analyses in SE patients detect infectious pathogens frequently. These findings and the fact that pathologic CSF findings on a chemical and/or cellular level in the absence of systemic infections call for rigorous screening to confirm or exclude infectious or autoimmune encephalitis and should not be withheld. More consistent and emphasized recommendations to perform lumbar puncture, especially in patients without plausible SE etiology are needed by international guidelines on SE management.

## Supplementary Information

Below is the link to the electronic supplementary material.

Supplementary file1 (DOCX 19 KB)

Supplementary file2 (TIFF 36,765 KB)

## Data Availability

Anonymized data are available from the corresponding author and will be shared on reasonable request from any qualified investigator.
