# Supplementary material for: Diagnostic yield of cerebrospinal fluid analysis in status epilepticus: an 8-year cohort study
Source: J Neurol. 2021 Mar 5;268(9):3325–36. doi: 10.1007/s00415-021-10447-3 (PMC8357741; doi:10.1007/s00415-021-10447-3)
Supplement: Supplementary file 1 — Supplementary file1 (DOCX 19 KB) [file 415_2021_10447_MOESM1_ESM.docx]

**Online Supplemental Table 1:** Detailed information regarding diagnostic workup using cerebrospinal fluid in patients with status epilepticus

| **Diagnostic workup from cerebrospinal fluid** | **n** | **% of patients with lumbar puncture** | **% of patients with lumbar puncture and persistent unknown etiologies** | |
| --- | --- | --- | --- | --- |
| **Chemical analyses** |  |  |  | |
| Lactate | 56 | 77.8 | 100 | |
| Lactate ratio CSF/serum | 51 | 70.8 | 84.2 | |
| Glucose | 56 | 77.8 | 100 | |
| Glucose ratio CSF/serum | 51 | 70.8 | 84.2 | |
| Protein | 56 | 77.8 | 100 | |
| Ferritin | 26 | 36.1 | 50.0 | |
| Immunoglobulins | 51 | 70.8 | 100 | |
| Oligoclonal bands | 51 | 70.8 | 100 | |
| **Cellular analyses** |  |  |  | |
| Leukozytes  (incl. differentiation of mono-/polynuclear) | 56 | 77.8 | 100 | |
| **Gram staining** | 50 | 69.4 | 84.2 | |
| **Bacterial cultures** | 50 | 69.4 | 84.2 | |
| **fungal cultures** | 6 | 8.3 | 84.2 | |
| **Search for protozoa** | 49 | 68.1 | 10.5 | |
| **Polymerase chain reactions** | 50 | 69.4 | 84.2 | |
| **Screening for autoantibodies** |  |  |  | |
| NMDA-R | 9 | 12.5 | 31.6 | |
| VGKC | 9 | 12.5 | 31.6 | |
| GAD | 9 | 12.5 | 31.6 | |
| **Screening for paraneoplastic antibodies** |  |  |  | |
| Anti-Hu (ANNA1) | Paraneoplastic antibodies were only screened in the serum | | |  |
| Anti-Yo (PCA1) |  |  |  |  |
| Anti-Ri (ANNA2) |  |  |  |  |
| Anti-CV2 (CRMP5) |  |  |  |  |
| Anti-MA1 (PNMA1) |  |  |  |  |
| Anti-MA2/TR (PNMA2) |  |  |  |  |
| Anti-amphiphysin |  |  |  |  |

CSE = cerebrospinal fluid; NMDA-R = N-methyl-D-aspartate-receptor; VGKC = voltage-gated-kalium-channels; GAD glutamic acid decarboxylase; ANNA = antineuronal nuclear antibody; PCA = principal component analysis; CRMP = collapsin response mediator protein; PNMA = paraneoplastic antigen MA
