# Supplementary figures and images for: Diagnostic yield of cerebrospinal fluid analysis in status epilepticus: an 8-year cohort study
Source: J Neurol. 2021 Mar 5;268(9):3325–36. doi: 10.1007/s00415-021-10447-3 (PMC8357741; doi:10.1007/s00415-021-10447-3)

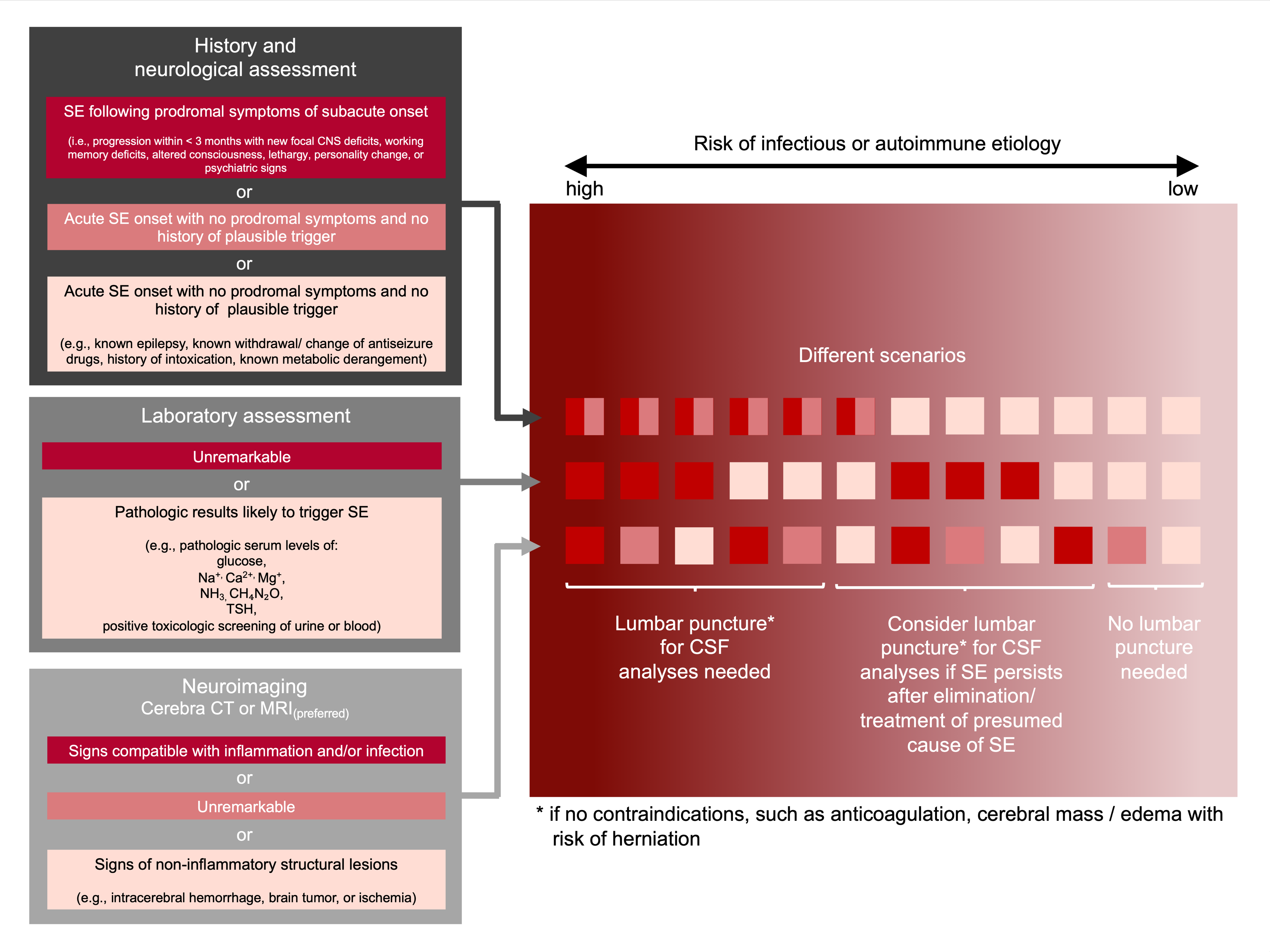

Supplement: Supplementary file 2 — Supplementary file2 (TIFF 36,765 KB) [file 415_2021_10447_MOESM2_ESM.tiff]
